# Dual Role of Small Noncoding RNA and Hfq in Bacterial DNA Compaction: A New Perspective on Nucleoid Architecture

**DOI:** 10.1021/acsomega.6c02202

**Published:** 2026-05-18

**Authors:** Gabriela Mistygacz, Satavisha Mukherjee, Jijo Easo George, Frank Wien, Indresh Yadav, Johan R. C. van der Maarel, Véronique Arluison

**Affiliations:** † Laboratoire Léon Brillouin, UMR 12 CEA/CNRS, Bâtiment 563, Site de Saclay, Gif-sur-Yvette 91191, France; ‡ Department of Physics, 231530 Indian Institute of Technology Bhubaneswar, Argul, Odisha 752050, India; § Department of Physics, 37580 National University of Singapore, Singapore 117542, Singapore; ∥ 55536 Synchrotron SOLEIL, L’Orme des Merisiers Saint Aubin, Gif-sur-Yvette F-91410, France; ⊥ Université Paris Cité, UFR SDV, 35 Rue Hélène Brion, Paris 75013, France

## Abstract

The spatial organization of bacterial chromosomes is commonly attributed to nucleoid-associated proteins that compact DNA through bending and bridging interactions. Yet, the possible structural role of RNA in this process remains largely unexplored. Here we show that a small noncoding RNA (sRNA), DsrA, directly modulates DNA organization through its interaction with the amyloidogenic C-terminal region (CTR) of the nucleoid associated protein and RNA chaperone Hfq. Using nanofluidic confinement, we find that the Hfq–CTR strongly compacts λ-DNA, whereas addition of sRNA partially suppresses this condensation despite simultaneous association of DNA and RNA with the CTR peptide. Synchrotron radiation circular dichroism (SRCD) spectroscopy reveals that the CTR, in the presence of sRNA, induces a conformational rearrangement of AT-rich DNA consistent with altered base stacking and increased stiffness. Passive particle-tracking microrheology shows that DNA–CTR assemblies form heterogeneous viscoelastic gels. Addition of sRNA increases the elastic modulus and shortens the relaxation time, indicating a more connected yet dynamically responsive network. When interpreted together with the nanochannel measurements, these findings reveal a decoupling between local DNA compaction and bulk mechanical rigidity: sRNA reduces DNA collapse at the molecular scale while enhancing connectivity of the DNA–peptide network at the mesoscale. We propose that sRNAs can regulate nucleoid architecture not only through gene regulation but also by directly tuning DNA mechanics via protein-mediated cross-linking. These findings identify sRNA as an active structural component of bacterial chromosomal organization and reveal a multiscale mechanism by which RNA-protein interactions control the physical state of the bacterial genome.

## Graphical abstract

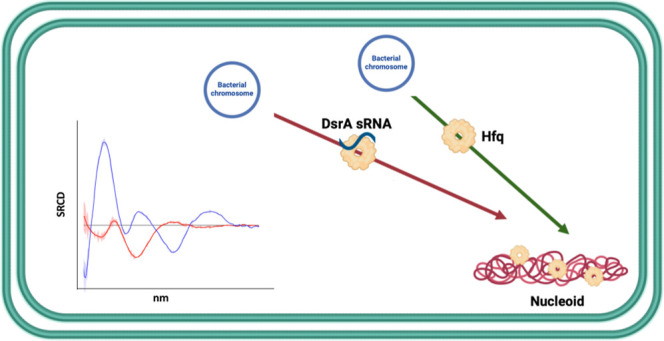

## Introduction

Bacterial genomic DNA is organized within a membrane-free region of the cell referred to as the nucleoid.^1^ The chromosomal circular DNA of *Escherichia coli* K12 is approximately 4.6 Mbp, which corresponds to a contour length of about 1.6 mm. Without structural constraints, this DNA would occupy a space much larger than the bacterial cell itself. Despite its considerable length, the DNA is thus tightly packed into a region within the cell with dimensions of approximately 0.5 μm in diameter, giving it a total volume of roughly 0.2 μm^3^, a volume that remains more or less constant throughout the cell cycle.^2–4^ Primary contributors to the compactness of bacterial DNA are nucleoid-associated proteins (NAPs).^4–6^ These passive DNA binders are believed to compact DNA through mechanisms such as bending, bridging, or clustering.^7^ Key DNA-bending proteins in *E. coli* include Fis (Factor for Inversion Stimulation), HU (Histone-like protein), and IHF (Integration Host Factor). DNA-bridging proteins such as H-NS (histone-like nucleoid-structuring protein) and Hfq (Host Factor Qβ) also contribute to DNA compaction.^8,9^ Both H-NS and Hfq preferentially bind to AT-rich regions and can form nucleoprotein filaments, which bridge DNA strands and lead to loop formation.^10^ Self-associating proteins indeed create a dynamic network that cross-links DNA segments, causing the DNA–protein complex to behave like a gel-like network.^11–13^ Despite forming a highly organized DNA–protein scaffold, the nucleoid is highly dynamic and retains sufficient flexibility to allow DNA accessibility and mobility, consistent with our recent work demonstrating the coexistence of compaction and functional accessibility.^14,15^ Overall, the bacterial nucleoid is commonly depicted as a cluster of supercoiled DNA loops organized around a scaffold of NAP proteins, forming macrodomains.^16,17^ Additionally, recent studies highlight macromolecular crowding as a key determinant of nucleoid spatial organization, with further stabilization provided by multivalent cations that neutralize the DNA’s negative charge.^13,18,19^

Hfq is an important protein involved in bacterial DNA compaction. This protein, primarily described as an RNA chaperone,^20^ is a central regulator of RNA metabolism and plays a multifaceted role in gene expression control.^21–23^ Hfq plays a vital role in facilitating the function of many small noncoding RNAs (sRNAs) in Gram-negative bacteria,^24^ particularly by enhancing the rate of sRNA/mRNA annealing to control translation efficiency when stresses occur.^25,26^ Hfq binding is also crucial for stabilizing many sRNAs.^27^ Structurally, *E. coli* Hfq forms a hexameric torus, referred to as the Sm-core.^28^ This corresponds to the N-terminal region of Hfq (NTR, residues 1–65), while six flexible C-terminal regions (CTR, residues 66–102) extend outward from the Sm ring.^29^ The Sm-fold adopted by the NTR is characterized by six bent antiparallel β-sheets, each preceded by an N-terminal helix. The six β-sheets of individual subunits assemble into a donut-shaped architecture. This NTR region is primarily responsible for RNA binding and annealing, facilitating the interaction between sRNAs and mRNAs.^30^ One of the surfaces of the Sm ring, where the N-terminal α-helices are located, is referred to as the proximal face, while the opposite side is known as the distal face.^31,32^ Each of these NTR surfaces engages RNA with distinct specificities and binding affinities.^31–33^ The proximal face interacts primarily with polyU sequences, typically found in the Rho-independent terminator regions of sRNAs.^34^ In contrast, the distal face recognizes A-rich triplet motifs.^32^ Since the Shine–Dalgarno sequence fits well within this A-rich sequence, Hfq typically binds mRNAs to its distal face, while sRNAs are bound to the proximal face. Note that the Shine–Dalgarno sequence is purine-rich (A/G-rich), and the distal face of Hfq preferentially binds A-rich sequences, including extended ARN motifs (A = adenine, R = purine, N = any nucleotide) which are commonly present in SD regions of mRNAs.^32,35,36^ Among the diverse sRNAs, two classes have been described. Class I sRNAs, which feature a poly­(U) tail that attaches to the proximal face of Hfq, usually along with a UA-rich motif that binds to the lateral rim, and Class II sRNAs, which also have a poly­(U) tail but contain an A-rich motif instead of a UA-rich motif, interacting with the distal face of Hfq.^24,37^

Hfq contains a C-terminal region tail, in addition to the NTR torus. The length of this CTR tail varies across species. The function of the CTR remained poorly understood until recent studies uncovered its propensity to form amyloid-like structures in *E. coli*.^38,39^ The amyloidogenic CTR does not appear to be essential for most sRNA-mediated regulation but contributes to RNA binding.^40^ Furthermore, it is critical for Hfq’s interaction with bacterial membranes, for RNA condensate formation and DNA shaping.^23,41–43^ Recent studies have revealed the structural basis of Hfq’s C-terminal extension in DNA compaction, identifying an amyloid module that uniquely bridges and compacts multiple DNA molecules.^44^ The acidic tip at the very end of the CTR is also believed to mimic nucleic acids, providing a competition with RNA and enhancing Hfq specificity.^45^ Note that while full-length Hfq binds poly­(A) RNA with high affinity,^46^ this property does not appear to extend to the isolated C-terminal region (CTR), which instead interacts with A-rich sequences without specific recognition of continuous 3′ poly­(A) tails.

Building on the observation of the dual function of the Hfq–CTR in RNA metabolism and DNA shaping, the present study investigates how an sRNA influences DNA shaping. Understanding this function of Hfq at the nucleoid interface could reveal new bacterial regulatory mechanisms, beyond current eukaryotic-based models of DNA compaction.

## Experimental Section

### Chemicals

Unless otherwise stated, all chemicals were purchased from Sigma-Aldrich or Thermo Fisher Scientific.

### DNA Compaction Analysis

To investigate how Hfq influences DNA compaction in the presence and absence of a small noncoding RNA, we selected DsrA as a representative type I sRNA. The ability of DsrA to cobind with both Hfq and DNA was established previously.^47^ Specifically, we used a fragment of DsrA, referred to as DsrA_core_,^26^ which comprises the first 37 nucleotides of the full-length 87-nucleotide RNA and retains the essential stem-loop 1 and Hfq-binding region.^48^ This shortened form preserves key double-stranded regions required for base-pairing with target mRNAs,^48^ particularly *rpoS*, while maintaining the ability to interact with Hfq and nucleic acids. Compared to full-length DsrA, DsrA_core_ offers enhanced stability and simplicity, making it widely used in in vitro studies as a minimal model to investigate the structural and functional aspects of Hfq–sRNA interactions.^26,40^ DsrA_core_ RNA oligonucleotide was synthesized by Eurogentec (Belgium) and its sequence was: AACACAUCAGAUUUCCUGGUGUAACGAAUUUUUUAAG.

Full length Hfq was expressed and prepared as described previously.^9,49^ Wild-type λ-DNA consisting of 48,502 base pairs (corresponding to a contour length of 16.5 μm) was obtained from New England Biolabs (Ipswich, MA, USA). To dissociate natural concatemers, DNA samples were heated to 333 K for 10 min and rapidly cooled to 295 K. Mixtures of Hfq, DNA, and DsrA_core_ were prepared in TE buffer (10 mM Tris–HCl, pH 8.0, 1 mM EDTA), with a final DNA concentration of 0.3 mg/L.

Nanofluidic devices were fabricated using polydimethylsiloxane with enhanced elastic modulus (X-PDMS), following established protocols.^9,50,51^ Each device contained rectangular nanochannels measuring 90 μm in length and approximately 120 × 130 nm in cross-section (referred to as the 125 nm system), with a dimensional tolerance of ±5 nm. DNA molecules were loaded into the nanochannels by electrophoresis.^9^ Upon cessation of the electric field, the molecules equilibrated within 2 min.

The conformational state of bacteriophage λ-DNA was examined by fluorescence microscopy, allowing visualization of DNA stretching and collapse in the presence of Hfq. DNA molecules were covalently labeled with MFP 488 dye at a ratio of one dye molecule per 50 base pairs, as determined by UV spectrometry (Mirus Bio, Madison, WI, USA). No antiphotobleaching agent was used in these preparations. The thus stained DNA maintains fluorescence and structural integrity under high ionic strength conditions. DNA–protein mixtures were prepared in TE buffer and confined within 125 nm-wide nanochannels, enabling quantitative measurement of DNA extension along the channel axis.

DNA visualization was performed using a Nikon Eclipse Ti inverted fluorescence microscope equipped with a 200 mW/488 nm diode laser (Omicron, Germany), an appropriate filter set, and a 100× oil immersion objective lens (numerical aperture 1.49). Time-lapse image sequences were captured at 10 frames per second with 100 ms exposure using an EMCCD camera (Andor iXon X3). The average extension of DNA molecules along the nanochannel was determined using a metric ruler.

### Synchrotron Radiation Circular Dichroism (SRCD)

Synchrotron radiation circular dichroism (SRCD), an electronic absorption spectroscopy, measures how chiral molecules in a sample interact with circular polarized light and is widely used to study the structure of biological macromolecules in solution. Compared to conventional CD, synchrotron radiation circular dichroism offers an extended wavelength range down to the vacuum ultraviolet (VUV), thereby increasing the spectral information content and improving structural insights.^42,52^ This extension is however limited to 168 nm for hydrated samples due to strong water absorption in the far-UV.

The sample analyzed consisted of the amyloid Hfq–CTR peptide^39^ bound to double stranded (ds) DNA sequences (dA/dT)_59_ or F11 sequence identified previously as Hfq interactors.^47,49,53^ The choices of these DNA sequences were made because Hfq and its CTR have high affinity for A-rich sequences.^54^ The C-terminal region of Hfq, corresponding to residues 64–102 of the full-length protein, was chemically synthesized (Proteogenix, France). The CTR peptide sequence was: SRPVSHHSNNAGGGTSSNYHHGSSAQNTSAQQDSEETE and it was prepared at a concentration of 20 mg/mL according to the protocol described by Fortas et al.^39^ dsDNA (dA/dT)_59_, and F11 DNA sequence^47,49,53^ were also synthesized by Eurogentec (Belgium). All NAs were dissolved in sterile, molecular biology-grade water at a final concentration of 1 mM (strand concentration) and relaxed for 2 min at 343 K followed by slow cool down back to 293 K before use. The formation of double stranded structure for DNA was checked on gel before use.

Complexes between DNA and Hfq–CTR peptides were prepared in water as described previously.^12,49^ Note that at the chosen concentration, the peptide shows self-buffering properties.^53^ Briefly, complexes were prepared in water and used at a final concentration of 1.8 mM for the peptide. The stoichiometry was 1 CTR per 4 base pairs for DNA, corresponding to 7.2 mM for DNA (expressed in base pair). The DsrA_core_ RNA concentration ranged from 1 to 50 μM expressed in strand (37 μM to 1.85 mM in nucleotides). The concentration was higher for DNA due to Hfq lower affinity compare to RNA.^54,55^ Since salts do not significantly affect the affinity of the CTR for DNA,^53^ we chose to omit additional salts in our SRCD preparations to obtain a broader spectral range (170–320 nm), which is crucial for accurately determining the structural features of nucleic acids.^56,57^ This procedure corresponds to the one used previously for the accumulation of references data set (https://genesilico.pl/nacddb/,^58^). Samples were analyzed after 3 weeks to allow the peptide self-assembly on DNA, that is not instantaneous.^49^

SRCD measurements were performed at the DISCO beamline at SOLEIL Synchrotron, following the procedure outlined by Malabirade et al.^49^ (proposals 20241127 and 20242209). Approximately 4 μL of sample was placed into a 33 μm path length CaF_2_ circular cell. Spectra were acquired in triplicate at 1 nm intervals with an integration time of 1.2 s, covering the range from 180 to 320 nm. Calibration of amplitude and wavelength positions was carried out using (+)-camphor-10-sulfonic acid (CSA). Data analysis, including averaging, baseline subtraction, smoothing, scaling, and spectral summation, was performed using CDtoolX.^59^ The resulting spectra are presented in millidegrees (mdeg) versus nanometers (nm), with the same molar ratios maintained across all samples. Due to the nature of the absorption, spectra of mixed samples (polynucleotides + peptides) could not be standardized to Δε.

### Passive Microrheology (Particle Video Tracking)

Three distinct sample formulations were prepared for comparative microrheological analysis. The CTR stock was prepared at a concentration of 30 mg/mL (or 7.792 mM). The control sample consisted of 25.4 mM (in base pairs) (dA/dT)_59_ DNA without CTR or sRNA, corresponding to a DNA/CTR stoichiometry of 1:0. The second sample was a DNA–CTR binary complex containing 25.4 mM DNA and 6.3 mM CTR, yielding a 4:1 DNA/CTR stoichiometry, with no sRNA present. The third sample was a DNA–CTR–sRNA complex with the same (4:1) DNA/CTR stoichiometry (25.4 mM DNA and 6.3 mM CTR), supplemented with 1 μM DsrA_core_ RNA. The DNA–CTR and DNA–CTR–sRNA complexes were allowed to equilibrate for approximately 2 weeks prior to measurements. All samples were spiked with polystyrene microspheres (Polysciences, Warrington, PA) of diameter 2.07 ± 0.05 μm at a final concentration of 0.001 wt %. A 25 μL sample was confined within a Bio-Rad Frame-Seal chamber (9 mm × 9 mm) between a glass slide and a coverslip to prevent evaporation and minimize convective drift. Particle tracking microrheology experiments were performed using an Olympus IX73 inverted microscope equipped with a 40× long working-distance objective (NA = 0.6, WD = 3.0–4.2 mm) at an ambient temperature of 296 K. The focal plane was positioned midway between the slide and coverslip at their maximum separation to minimize wall effects. Trajectories of 25–30 beads at randomly selected locations, separated by approximately 100 μm, were recorded using a CMOS camera (Basler acA1440–220 μm) at a frame rate of 100 fps. Video sequences of 3–5 min duration were analyzed using MATLAB (MathWorks, Natick, MA), with particle trajectories extracted using public domain tracking software (http://site.physics.georgetown.edu/matlab/). Subsequent data analysis was performed with home developed software scripts written in MATLAB code. The pixel resolution (0.088 μm/pixel) was calibrated using a metric ruler. Instrumental noise was assessed by tracking immobilized beads adsorbed onto the glass slide, which exhibited a mean-square displacement of approximately 10^–5^ μm^2^. This value sets a lower detection limit for the creep compliance of *J* ≈ 0.02 m^2^/N.

### Theoretical Basis

The bacterial nucleoid can be viewed as a polyelectrolyte polymer network, where nucleoid-associated proteins compact DNA through multivalent binding and cross-linking, giving rise to viscoelastic behavior and mesoscale mechanical connectivity. Here, we focus on the nucleoid, a nucleic acid-binding protein Hfq, and its interactions with small RNAs, probing their impact on nucleoid architecture. RNA/Hfq interactions introduce additional multivalency that can reorganize DNA–protein assemblies. Hfq intrinsically disordered or amyloidogenic domains simultaneously engage DNA and RNA, enabling RNA to redistribute, compete with, or reinforce cross-links. In this framework, RNA acts as an active structural component that modulates nucleoid organization through phase-like assembly and gelation, decoupling local DNA condensation from network mechanics and revealing a multiscale mechanism for Hfq–RNA-mediated chromosomal control.

## Results

### Hfq-Induced DNA Compaction and Its Modulation by sRNA

Representative fluorescence images of the wild type DNA substrates without Hfq and crowded by various concentrations of Hfq, as well as 1 μM Hfq mixed with 1 μM sRNA are shown in Figure 1A. Due to the relatively low staining ratio of one dye per 50 base pairs and potential effects of photobleaching, the molecules appeared heterogeneously stained. The mean end-to-end distance along the channel is shown in Figure 1B (average of 30 independently measured molecules). At low Hfq concentrations, DNA exhibited a nearly constant extension of 8.7 ± 0.1 μm, corresponding to approximately 53% of its contour length. When the concentration of Hfq exceeded a critical threshold, DNA underwent a sharp transition into a compacted state, consistent with previous observations for Hfq–DNA complexes in wider nanochannels.^9,43^ At an Hfq hexamer concentration of 1 μM, compacted DNA displayed a reduced extension of 1.2 ± 0.2 μm. When an equimolar amount of DsrA_core_ was added (Hfq/DsrA_core_ = 1:1, DsrA_core_ expressed in strand concentration), DNA did not fully compact but maintained a mean extension of 3.7 ± 0.6 μm. These results indicate that the interaction between Hfq and DsrA_core_ diminishes Hfq’s ability to promote DNA condensation. Previously, we have investigated the compaction of DNA by full-length Hfq, Hfq–CTR, and Hfq–NTR in the bulk phase as well as inside nanochannel systems.^9,43^ Hfq condenses DNA similarly to Hfq–CTR. For ease of reference, some results are reproduced in Supporting Information Figure S1. In the case of Hfq–NTR, no condensation either in the channel systems or in the bulk phase is observed. Furthermore, the critical concentration of Hfq (and Hfq–CTR) for condensation inside channels is about an order of magnitude lower than in bulk solution.

**Figure 1:**
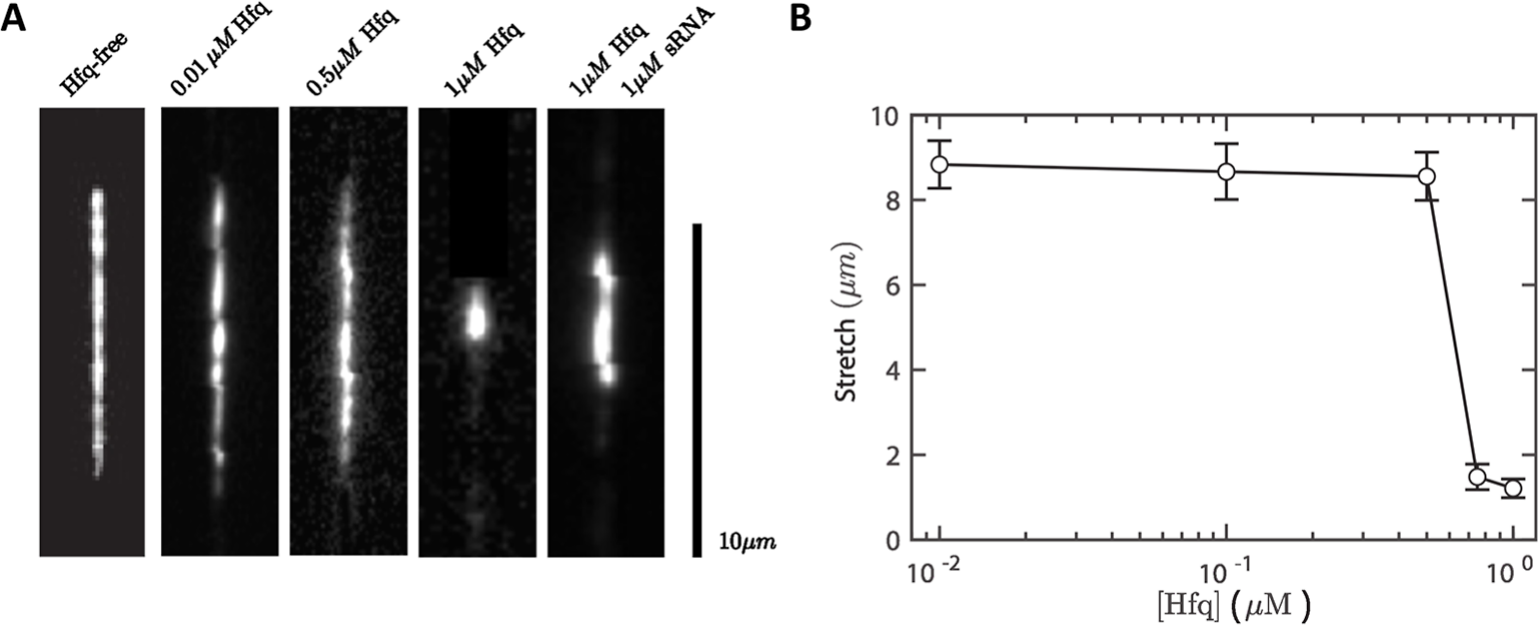
(A) Fluorescence images wild-type λ-DNA confined to a 125 nm channel. The DNA molecules are compacted by Hfq and sRNA (DsrA_core_) with designated concentrations. (B) Mean end-to-end distance of the confined λ-DNA molecules versus the concentration of Hfq. sRNA concentrations below 1 μM produced little to no detectable DNA compaction or stretching relative to the Hfq-free state. Conversely, increasing DsrA_core_ concentration would further deviate from physiological levels.^60^

### sRNA-Dependent Structural Remodeling of DNA Revealed by SRCD

Because CTR drives DNA compaction, we then focused on a reductionist system to uncover mechanistic principles that are difficult to resolve with the full-length Hfq/DNA complex, owing to full Hfq DNA melting activity of its NTR, and the structural heterogeneity of the long viral DNA. Note that the use of the full-length protein is also limited by its low solubility, particularly in the presence of DNA, which precludes reliable SRCD measurements under these conditions.

As shown in Figure 2, at a high DsrA_core_ concentration (50 μM; red spectrum), the SRCD signal of DNA (blue spectrum) exhibited a distinct spectral inversion, whereas no inversion was observed without DsrA_core_ and at a low DsrA_core_ concentration (1 μM; green spectrum). Because the sRNA contributes negligibly to the SRCD signal in this spectrum, the inversion reflects a conformational change in DNA. The concentration-dependent response confirmed that this is not an experimental artifact due to subtraction. It should be noted that the concentrations used in SRCD are not directly comparable to those in nanochannel imaging due to the absence of confinement. In bulk solution, condensation requires Hfq concentrations that are approximately an order of magnitude higher.^9,43^ Nevertheless, this effect was observed at a CTR/sRNA ratio of 1:1, in agreement with the fluorescence experiments. DNA alone was included as a control. DsrA alone shows no detectable effect on the DNA spectrum. Although the sRNA does have an SRCD signal, it is not observable under our experimental conditions due to its low concentration; therefore, we can only observe changes in the structure of DNA due to the presence of DsrA_core_ in the presence of Hfq, not DsrA_core_ itself. At higher concentrations, this signal would become detectable.^40^

**Figure 2:**
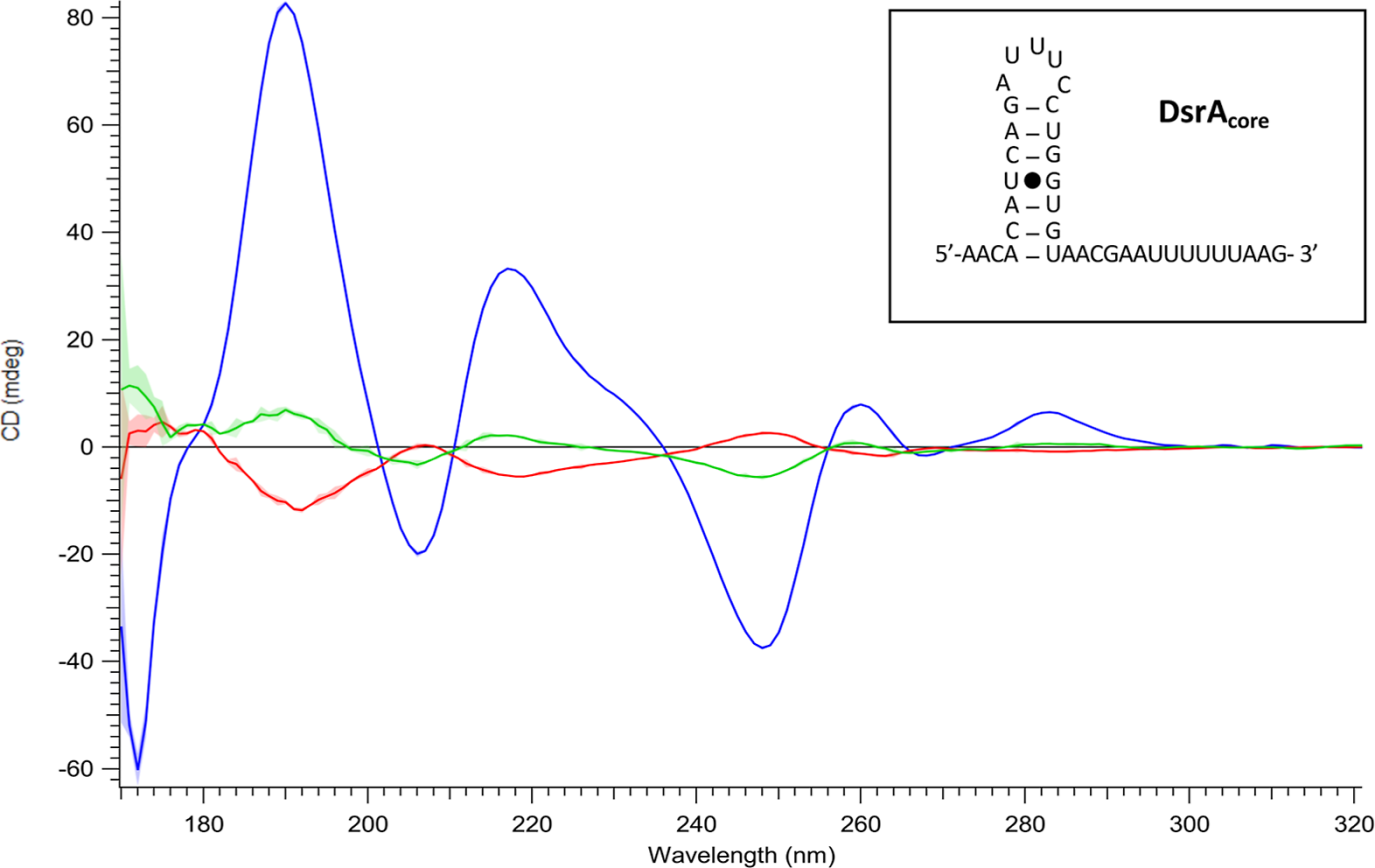
Effects of Hfq–CTR on DNA (dA/dT)_59_ in the presence of DsrA_core_. Inset: DsrA_core_ secondary structure. SRCD-spectral differences and inversions were observed upon high or low DsrA_core_ concentration. By subtracting the spectrum of CTR + DNA from CTR + DNA + DsrA_core_, spectral changes become evident indicating structural changes of the (dA/dT)_59_ DNA structure. Blue: DNA alone (DNA (dA/dT)_59_) exhibits a specific signature in CD, different from classical B-form DNA.^58^ Small spectral differences that are difficult to discern in original spectra (see Supporting Information Figure S2) can be effectively highlighted using a difference spectrum, where subtraction of a reference spectrum enhances weak or overlapping features (see Supporting Information Figure S2);^61^ Red: CTR + DNA + DsrA_core_ 50 μM (subtracted from CTR + DNA (dA/dT)_59_); green: CTR + DNA + DsrA_core_ 1 μM (subtracted from CTR + DNA (dA/dT)_59_). As shown for DsrA_core_ at 50 μM we observed a complete inversion of the spectra after spectral subtraction, indicating chirality changes of the DNA (dA/dT)_59_ structure in comparison to DNA + DsrA_core_ at 1 μM, which means that DsrA has a meaningful impact on the DNA (dA/dT)_59_. Colored contours represent the standard deviation obtained from three consecutive acquisitions including the subtracted baseline.

To test whether this effect is sequence-independent, we repeated SRCD analysis using the F11 DNA fragment, a natural AT-rich Hfq target (241 nucleotides-long).^47^ AT-rich sequences are frequently present in promoters.^62^ As shown in Figure 3, the CTR-F11-DsrA_core_ complex also exhibited a spectral inversion relative to unbound DNA, confirming that Hfq’s CTR in the presence of the type I sRNA drives a similar structural rearrangement for a natural DNA AT-rich sequence.

**Figure 3:**
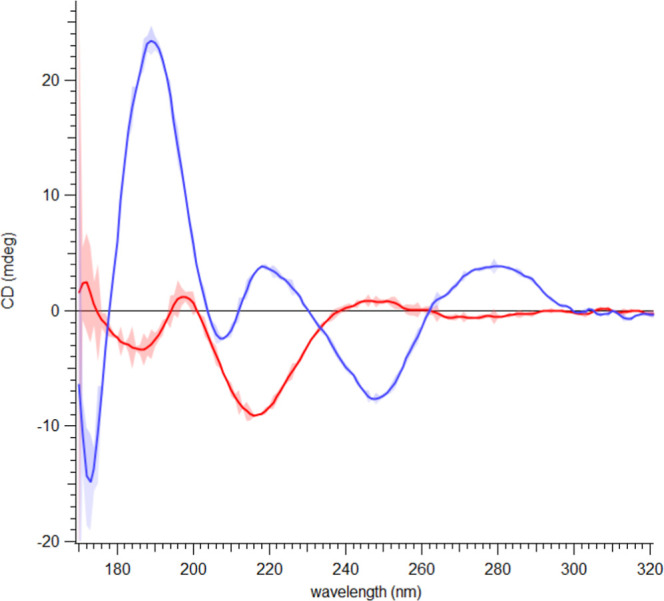
Same as in Figure 2 but with F11 DNA. Blue: DNA alone; red: CTR + DNA + DsrA_core_ 50 μM (subtracted from CTR + DNA). The CD spectrum of our sample displays the characteristic signature of the X-form,^63^ with a negative band at ∼280 nm and a positive band at ∼205 nm.

Our results thus demonstrate that Hfq, through its C-terminal region, exerts a strong influence on DNA structure and compaction that can be affected by the presence of a noncoding RNA. Precisely, while Hfq alone (due to its CTR) induces pronounced DNA condensation, the addition of a sRNA (here DsrA) attenuates this effect. The cobinding of DsrA_core_ and DNA to Hfq indicates that the observed modulation is not caused by direct competition for binding sites, but instead arises from structural rearrangements within the Hfq–nucleic acids complex. SRCD spectroscopy revealed a concentration-dependent inversion of DNA spectral signatures, indicative of alterations in base stacking and/or helical chirality.

### Viscoelastic Properties and Heterogeneity of DNA–CTR Networks

To examine how interactions between CTR and sRNA modify the mechanical response of DNA, microrheology experiments were performed. The viscoelastic properties of the DNA–CTR dispersions were probed based on video tracking of embedded colloidal beads. This approach enables measurements of the local viscoelastic response, thereby allowing characterization of spatial heterogeneity arising from CTR-mediated assemblies on DNA.^12^ Moreover, the measurements probe the system under equilibrium, thermally driven, low-shear conditions, so that DNA density remains unperturbed on length scales larger than the probe particle, as the measurements rely solely on thermal fluctuations.^64^ The formation of a physical gel is readily identified by a plateau in the bead mean-square displacement at long lag times.^12,64^ From the recorded bead trajectories, probability distributions of displacements in the *x*- and *y*-directions were determined over a range of lag times *t*. Gaussian functions were fitted to these distributions to extract the mean-square displacement ⟨Δ*x* ^2^(*t*)⟩. The variances obtained along the two orthogonal directions were averaged, such that ⟨Δ*x* ^2^(*t*)⟩ represents the one-dimensional mean-square displacement. The time-dependent creep compliance was calculated using *J*(*t*) = 3π*a*⟨Δ*x* ^2^(*t*)⟩/*k*_B_ *T*, where *k*_B_ *T* denotes the thermal energy and *a* is the bead radius.^64^

Figure 4 presents the mechanical properties of control DNA (25.4 mM (dA/dT)_59_ in base pairs) and DNA–CTR assemblies at a fixed stoichiometric ratio (DNA/CTR = 4:1). Tracer particles embedded in control DNA exhibit a creep compliance that increases linearly with lag time (Figure 4a), consistent with purely viscous flow behavior described by *J*(*t*) = *t*/η with a static viscosity η = 1.25 ± 0.01 mPa s. In contrast, the DNA–CTR assemblies display a qualitatively different temporal response. As shown in Figure 4b, the creep compliance initially grows at short times and subsequently approaches a time-independent plateau at long lag times, indicative of elastic confinement of tracer beads within a percolated network. Notably, both the magnitude and the onset time of the plateau vary among individual tracer particles, indicating spatial heterogeneity in the local mechanical properties of the gel. Control measurements confirm that this variability is independent of tracer bead size and instead arises from intrinsic inhomogeneities in the viscoelastic response of the DNA–CTR network.

**Figure 4:**
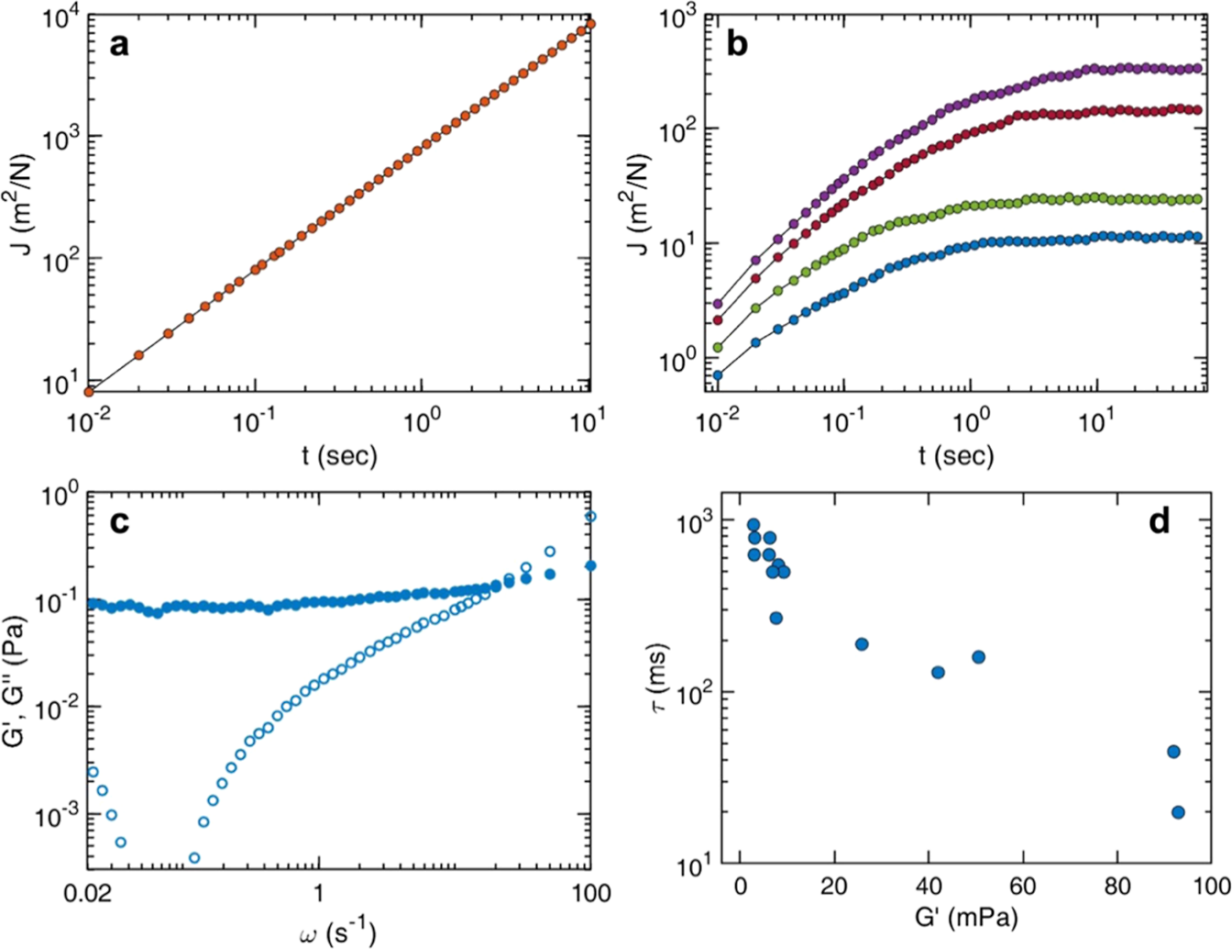
Viscoelastic response of DNA–CTR assemblies: (a) creep compliance *J*(*t*) of tracer particles in control DNA (25.4 mM in base pairs), displaying purely viscous flow. (b) Representative *J*(*t*) curves for DNA–CTR assemblies (DNA/CTR = 4:1), measured at four randomly selected bead locations within the sample, showing long time plateaus that indicate elastic confinement and mechanical heterogeneity within the network. (c) Storage (*G*′) (closed symbol) and loss (*G*″) (open symbol) moduli derived from the lowest *J*(*t*) revealing solid-like behavior at low frequencies. (d) Low-frequency limit of *G*′ and the crossover relaxation time, τ = ω_c_^–1^ measured at 14 different locations.

To further quantify the mechanical response of the DNA–CTR assemblies, the storage (*G*′) and loss (*G*″) moduli were extracted from the creep compliance data using a one-sided Fourier transform in conjunction with the generalized Stokes–Einstein relation.^65,66^ This analysis assumes an incompressible continuum, no-slip boundary conditions at the tracer surface, and the validity of Stokes drag over the probed frequency range. Figure 4c presents representative frequency-dependent storage and loss moduli obtained from the lowest creep compliance in Figure 4b. At low frequencies, the storage modulus is approximately constant and exceeds the loss modulus (*G*′ > *G*″), indicating that elastic stress storage dominates the long-time mechanical response. With increasing frequency, the loss modulus increases and becomes comparable to or larger than the storage modulus, reflecting a crossover to viscous dissipation at shorter time scales. On these experimentally relevant time scales, viscous drag strongly outweighs inertial effects, and tracer bead motion is governed by the interplay of viscous dissipation, elastic confinement imposed by the DNA–CTR network, and thermal fluctuations. Under these conditions, particle dynamics are well described by effective harmonic confinement, leading to a mean-squared displacement ⟨Δ*x* ^2^(*t*)⟩ = *x*_0_^2^[1 – exp­(−*t*/τ)], where *x*_0_^2^ is the confinement amplitude and τ is the characteristic relaxation time associated with network rearrangements.^64^ Under these conditions, the frequency-independent storage modulus is directly related to the long-time creep compliance according to *G*′ = *K*_B_ *T*/(3π*ax*_0_^2^) = 1/*J*(*t* → ∞) .The loss modulus exhibits a linear dependence on frequency *G*″ = ωτ*G*′ and the crossover between elastic and viscous dominated response is defined by the characteristic frequency ω = τ^–1^. As observed for the creep compliance, both the storage (*G*′) and loss (*G*″) moduli vary spatially across the sample. This heterogeneity was quantified by extracting the low-frequency plateau value of *G*′ and the associated crossover relaxation time τ = ω_c_^–1^, at multiple probe locations. The results are shown in Figure 4d for 14 different locations. The relaxation time varies by more than an order of magnitude, with longer relaxation times observed at locations where the storage modulus *G*′ is lower.

We next investigated how the incorporation of sRNA alters the mechanical response of DNA–CTR assemblies. The corresponding microrheological measurements are presented in Figure 5. Representative creep compliance curves *J*(*t*) obtained from tracer particles at four randomly selected positions within the sample (Figure 5a), display the same qualitative time dependence as observed for DNA–CTR assemblies in the absence of sRNA, confirming that gelation is preserved upon RNA addition. However, the minimum observed creep compliance is substantially reduced in the presence of sRNA, indicating an increase in the elastic storage modulus, *G*′ = 1/*J*(*t* → ∞) . The frequency-dependent viscoelastic moduli extracted from the lowest *J*(*t*) are shown in Figure 5b. To quantify the impact of RNA on network mechanics and heterogeneity, the low-frequency elastic plateau modulus *G*′ and the characteristic relaxation time, τ = ω_c_^–1^, were determined at multiple probe locations for both DNA–CTR and DNA–CTR–sRNA assemblies. Measurements from 23 probe locations are summarized in Figure 5c. A variation over an order of magnitude is observed in both the parameters with a longer relaxation time for locations with lower *G*′. We further compared the heterogeneity in mechanical response with and without sRNA. *G*′ and τ are characteristic parameters describing the gel’s response to thermal or other perturbations. Specifically, *G*′ reflects the elastic stiffness (i.e., resistance to deformation), whereas τ represents the characteristic stress relaxation time. The distributions in *G*′ and τ, shown as violin plots in Figure 6a,b, capture the full spread of measured values, where the width reflects local data density, the solid line denotes the median, and the dashed lines indicate the first and third quartiles.^58^ In both cases, the broad distributions indicate pronounced spatial heterogeneity, consistent with an inhomogeneous network formed by CTR-mediated DNA assemblies. Incorporation of sRNA systematically shifts these distributions, increasing the median storage modulus (*G*′) while reducing the median relaxation time (τ). This is indicative of a stiffer network with faster local stress relaxation. Despite these shifts, the distributions remain broad, showing that spatial heterogeneity is preserved. Overall, the mechanical response is shifted toward a stiffer and more dynamically responsive gel, demonstrating that small noncoding RNA enhances network stiffness without disrupting gelation.

**Figure 5:**
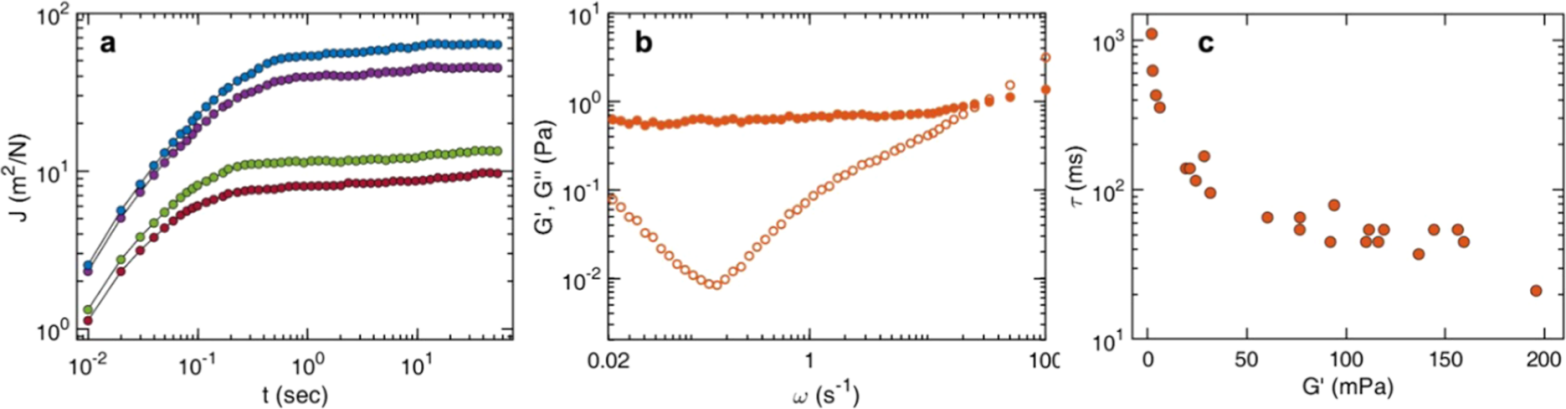
Viscoelastic response of DNA–CTR assemblies in the presence of 1 μM sRNA: (a) representative *J*(*t*) curves for DNA–CTR assemblies (DNA/CTR = 4:1), with 1 μM sRNA measured at four randomly selected bead locations within the sample, showing long time plateaus that indicate elastic confinement and mechanical heterogeneity within the network. (b) Storage (*G*′) (closed symbol) and loss (*G*″) (open symbol) moduli derived from the lowest *J*(*t*) revealing solid-like behavior at low frequencies. (c) Low-frequency limit of *G*′ and the crossover relaxation time, τ = ω_c_^–1^ measured at 23 different locations.

**Figure 6:**
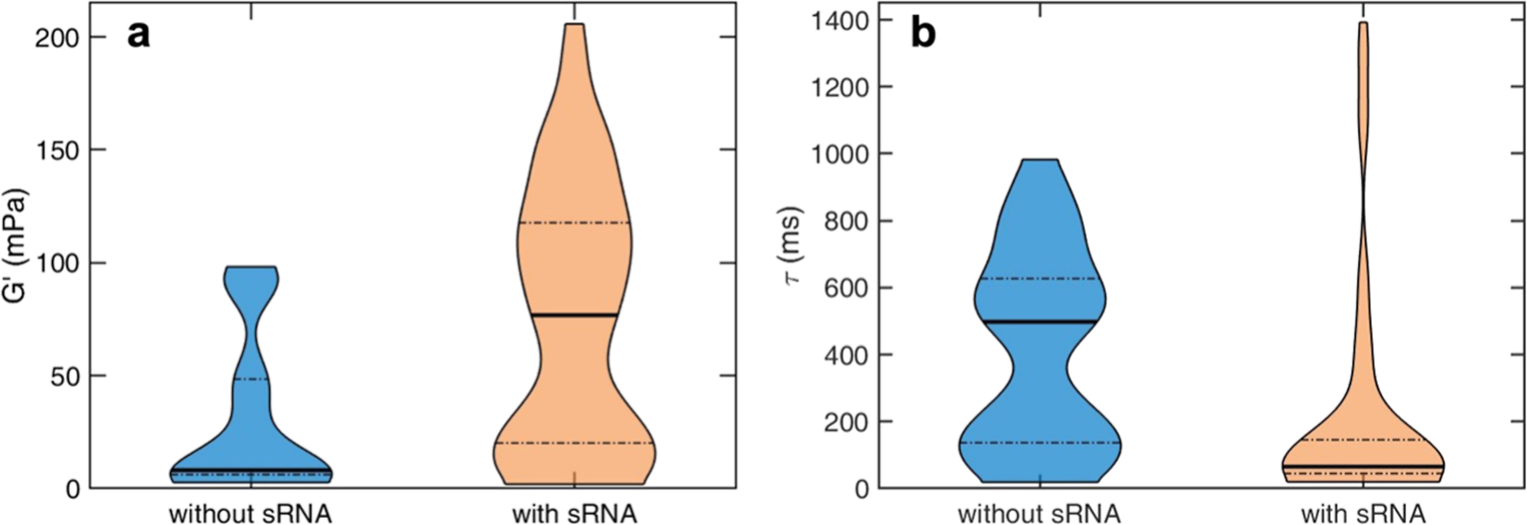
Violin plots showing the distributions of (a) the low-frequency elastic storage modulus, *G*′, and (b) the crossover relaxation time, τ, for DNA–CTR assemblies in the absence (cyan) and presence (orange) of 1 μM sRNA.

## Discussion

Nanochannel measurements show that the presence of sRNA decreases DNA compaction by full length Hfq (Figure 1). Because this compaction is driven by the CTR, this result indicates that sRNA binding attenuates CTR-mediated local DNA condensation. Although DsrA is known to bind the proximal face of Hfq,^67^ we hypothesized that the reduced DNA compaction observed in the presence of sRNA could stem from competition between DNA and RNA for Hfq binding. The DNA-binding surface of Hfq remains debated: crystallographic data indicate proximal binding,^68^ whereas solution analyses suggest interaction with the distal face.^47^ Gel electrophoresis analyses confirmed cobinding of Hfq with both DsrA and DNA,^47^ thereby ruling out competitive binding. The affinity of Hfq for DsrA_core_ (∼200 nM)^55^ is notably higher than that for AT-rich DNA sequences (micromolar range).^54^ Therefore, the observed decrease in DNA compaction is unlikely due to direct binding competition but may instead arise from structural effects induced by sRNA binding.

The SRCD spectral inversion relative to unbound DNA, confirm that Hfq’s CTR in the presence of type I sRNA promotes a structural rearrangement in AT-rich sequences. Typically, a CD spectrum inversion reflects a change in the chiral environment and in the conformation of DNA. For instance, in the canonical right-handed B-DNA form, a right-handed helix, a positive band is observed at ∼270–280 nm and a negative band at ∼245 nm, while in the left-handed Z-DNA form, the CD spectrum is nearly an inversion of that of B-DNA: e.g., a negative band near ∼290 nm, a positive band near ∼260 nm.^58^ Although an inversion of the CD spectrum might suggest a Z-form, this is unlikely in our case as Z-form DNA predominantly occurs in GC-rich sequences. Nevertheless, Z-form may occasionally form in AT rich sequences^69^ and the local stabilization of such a left-handed conformation by a protein cannot be entirely excluded.^70,71^ Alternatively, SRCD inversion may reflect a perturbation of base stacking and pairing without complete helical inversion. For instance, CD spectral inversions arising from the interaction of cationic gemini surfactants (used for cell transfection) with DNA have previously been reported.^72^ By analogy, the association between the Hfq–CTR and sRNA may elicit a comparable phenomenon. In particular, the histidine repeats within the Hfq CTR could, in the presence of RNA, mimic the behavior of the two imidazolium rings characteristic of gemini surfactants, thereby inducing a similar CD spectral response and spectral inversion.^72^

The CD changes observed here suggest switching of the AT repeats found in Hfq–sRNA-bound DNA, into an X-form, an extended right-handed helix where adenines in a syn conformation on antiparallel strands can form Hoogsteen base pairs.^63,73–75^ The CD spectrum of our sample indeed displays the characteristic signature of the X-form, with a negative band at 280 nm and a positive band at 210 nm. The X-form stiffens DNA and reduces its bendability.^76^ As a result, the DNA becomes less flexible and less prone to compaction, leading to an overall decrease in chromosomal condensation, thereby explaining the partial inhibition of compaction observed by fluorescence microscopy. Interestingly, the ribose sugars in X-DNA adopt a conformation closer to O4′-endo and C3′-endo than the C2′-endo conformation typical of B-DNA,^77^ and Hfq has previously been shown to favor sugar repuckering in DNA.^54^ Together, these observations suggest that the sRNA/Hfq complex modulates DNA mechanics at the structural level, providing a mechanistic basis for its anticompaction effect on the bacterial nucleoid.

The dual effect on DNA compaction and bulk mechanical rigidity likely arises because DNA and sRNA simultaneously interact with the CTR, redistributing CTR binding and thereby limiting tight DNA collapse. In contrast, microrheology reveals that the same sRNA addition results in a mechanically stiffer network. Within the framework of polymer network elasticity, the low-frequency storage modulus scales with the density of elastically active constraints as *G*′ = ν*k*_B_ *T* where ν is the number of cross-links per unit volume.^65,78^ The observed increase in *G*′ accompanied by a reduction in the characteristic relaxation time, indicates that sRNA enhances network connectivity by increasing the number of elastically active constraints (ν) while simultaneously reducing the effective strand length between elastically active cross-links. Thus, although sRNA suppresses local DNA compaction, it promotes the formation of a more highly connected and elastically efficient DNA–CTR network, resulting in a stiffer yet dynamically more responsive gel. This decoupling of compaction from elasticity highlights the role of sRNA as a mechanical regulator that modulates CTR-mediated cross-linking without disrupting gelation. Notably, the nanochannel and SRCD measurements primarily probe local DNA conformation and segmental structure, while microrheology captures the collective mechanical response of the assembled network. Together, these measurements show that sRNA modulates DNA–CTR interactions across different length scales, reducing local DNA condensation while simultaneously enhancing global network connectivity and mechanical robustness.

This work thus broadens our understanding of noncoding RNA-mediated regulation of DNA architecture. In eukaryotes, ncRNAs, particularly long noncoding RNAs (lncRNAs) and small RNAs (siRNAs, miRNAs), promote chromatin compaction. In almost all cases, ncRNAs act indirectly, by recruiting protein complexes that modify histones or the DNA methylation state.^79^ In contrast, our findings show that in bacteria, sRNA exerts an anticompaction effect, by directly modulating the activity of a nucleoid-associated protein Hfq, although this may slightly differ in vivo due to the use of a truncated form of the protein. This mechanism thus differs from the eukaryotic model in two key aspects and advances our understanding of ncRNA-mediated regulation of chromatin architecture.

This NAP-dependent modulation of DNA structure by sRNA may thus provide a mechanism for dynamic control of nucleoid architecture and gene expression. By altering the compaction efficiency of Hfq, sRNAs could regulate local DNA accessibility and transcriptional responsiveness to environmental stimuli. Note that a similar mechanism has been reported in bacteria where cruciform DNA structures and ncRNAs cooperate with HU to influence compaction, but in this case the sRNA promoted condensation.^80^ Hfq may function analogously, with sRNAs to fine-tune DNA conformation through indirect structural effects, to reduce it compaction (Scheme 1).

**Scheme 1:**
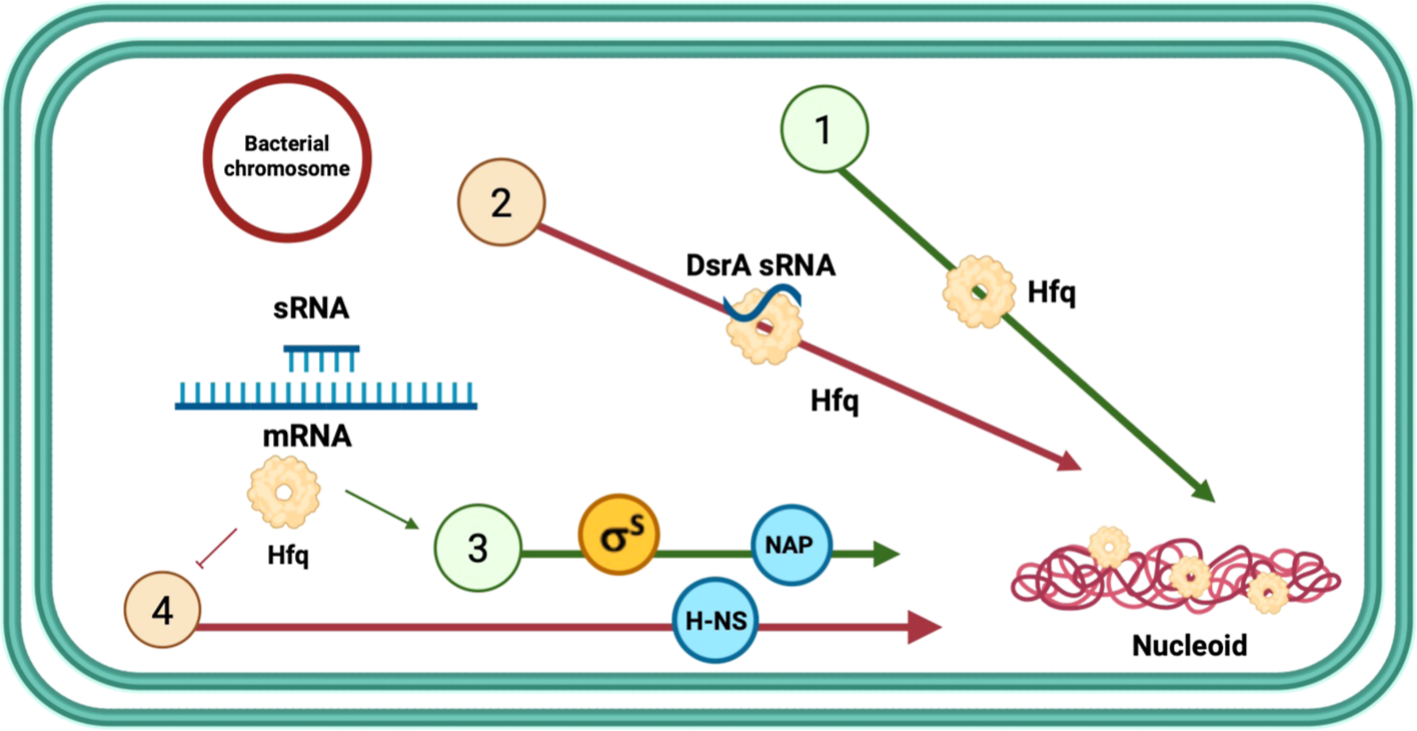
Model of Hfq- and DsrA-Dependent Regulatory Pathways Controlling DNA Compaction^a^

a Route 1 represents the direct Hfq-mediated DNA compaction pathway.^43^ Route 2 represents the newly discovered direct negative effect of DsrA on DNA compaction, which is dependent on Hfq. Route 3 illustrates an Hfq-mediated compaction pathway involving nucleoid-associated proteins (NAPs) expressed from promoters activated by the σ^S^ factor, whose own expression is regulated post-transcriptionally by sRNAs, including DsrA.^84^ Because this pathway is σ^S^-dependent, it is primarily active during the stationary growth phase. Route 4 depicts the DsrA-dependent indirect negative regulation of DNA compaction, which occurs through the control of *hns* mRNA translation. This process depends on DsrA sRNA and Hfq.^85,86^ The current work demonstrates that route 2 using sRNA enhances network connectivity in DNA–CTR assemblies, increasing elastic rigidity while promoting faster stress relaxation, consistent with a mechanically reinforced yet dynamically adaptable gel. In the scheme, RNAs are shown in blue; Hfq is depicted as a light-orange toroidal hexamer; the NAPs including H-NS as blue ellipses; chromosomal DNA as a purple line. Positive and negative routes are indicated by green and red arrows, respectively. Similarly, positive and negative regulations by Hfq/sRNA are indicated by green arrows and red T-shaped lines, respectively. The outer and inner membranes are shown in green.

Interestingly, in the nucleoid-riboid phase separation model,^1^ the bacterial cytoplasm is proposed to comprise two distinct regions: a DNA-rich nucleoid and a ribosome-rich “riboid,” separated by a functional interfacial zone. Within this framework, the nucleoid periphery is thought to serve as a structured interface where transcription is concentrated.^81^ NAPs, including Hfq, are proposed to contribute to the organization of this boundary. In this context, small regulatory RNAs (sRNAs) found within the nucleoid^82^ may further modulate NAP-DNA interactions, promoting DNA mobility while preserving overall structural cohesion.

## Conclusion

In conclusion, our findings reveal that sRNA binding modulates the structural state of DNA through Hfq, thereby reducing its compaction efficiency. Hfq contributes to DNA compaction via both direct and indirect mechanisms: directly, by bridging distant DNA segments, and indirectly, by regulating the expression of other nucleoid-associated proteins through its role in sRNA-mediated gene regulation (Scheme 1). Notably, DsrA sRNA targets multiple mRNAs, including *hns*, which encodes the H-NS NAP.^83^ By negatively regulating *hns* mRNA translation, DsrA indirectly reduces DNA compaction. Here, we further show that a class I sRNA (DsrA) also negatively influences DNA condensation through a direct pathway. These results reveal a previously unrecognized role for bacterial sRNAs in modulating DNA organization, extending their regulatory functions beyond sRNA–mRNA interactions to include the structural regulation of the nucleoid.

## Supplementary Material



## Abbreviations

NAP: nucleoid associated protein
sRNA: small noncoding RNA
(SR)­CD: (synchtrotron radiation) circular dichroism
NTR/CTR: N/C-terminal region
