# Supplementary material for: Dual Role of Small Noncoding RNA and Hfq in Bacterial DNA Compaction: A New Perspective on Nucleoid Architecture
Source: ACS Omega. 2026 May 18;11(21):31559–69. doi: 10.1021/acsomega.6c02202 (PMC13234807; doi:10.1021/acsomega.6c02202)
Supplement: Supplementary file 1 [file ao6c02202_si_001.pdf]

The Dual Role of Small Noncoding RNA and Hfq in Bacterial DNA Compaction: A New Perspective on Nucleoid Architecture

*Gabriela Mistygacz[a], Satavisha Mukherjee[b], Jijo Easo George[c], Frank Wien[d], Indresh Yadav[b], Johan R. C. van der Maarel[c] and Véronique Arluison[a,d,e]**

[a] *Laboratoire Léon Brillouin, UMR 12 CEA/CNRS, Bâtiment 563, Site de Saclay, 91191 Gif-sur-Yvette, France*

[b] *Department of Physics, Indian Institute of Technology Bhubaneswar, Argul-752050, Odisha, India*

[c] *Department of Physics, National University of Singapore, 117542, Singapore*

[d] *Synchrotron SOLEIL, L'Orme des Merisiers Saint Aubin, F-91410 Gif-sur-Yvette, France*

[e] *Université Paris Cité, UFR SDV, 35 Rue Hélène Brion, 75013 Paris, France; veronique.arluison@u-paris.fr*

* *Correspondence: veronique.arluison@u-paris.fr; 33 (0)169083282*

Supplementary figure S1 : ***(A)*** Relative extension R/L of T4–DNA inside 200 by 200 nm^2 channels and in T-buffer versus the concentration of Hfq (blue, squares), Hfq–CTR (red, circles) and Hfq–NTR (green, diamonds). ***(B)*** As in panel A, but for T4–DNA inside 150 by 250 nm^2 channels and in T-buffer with 30 mM KGlu. The dashed curves are drawn as an aid to the eye and the arrows denote the condensation thresholds [1, 2].

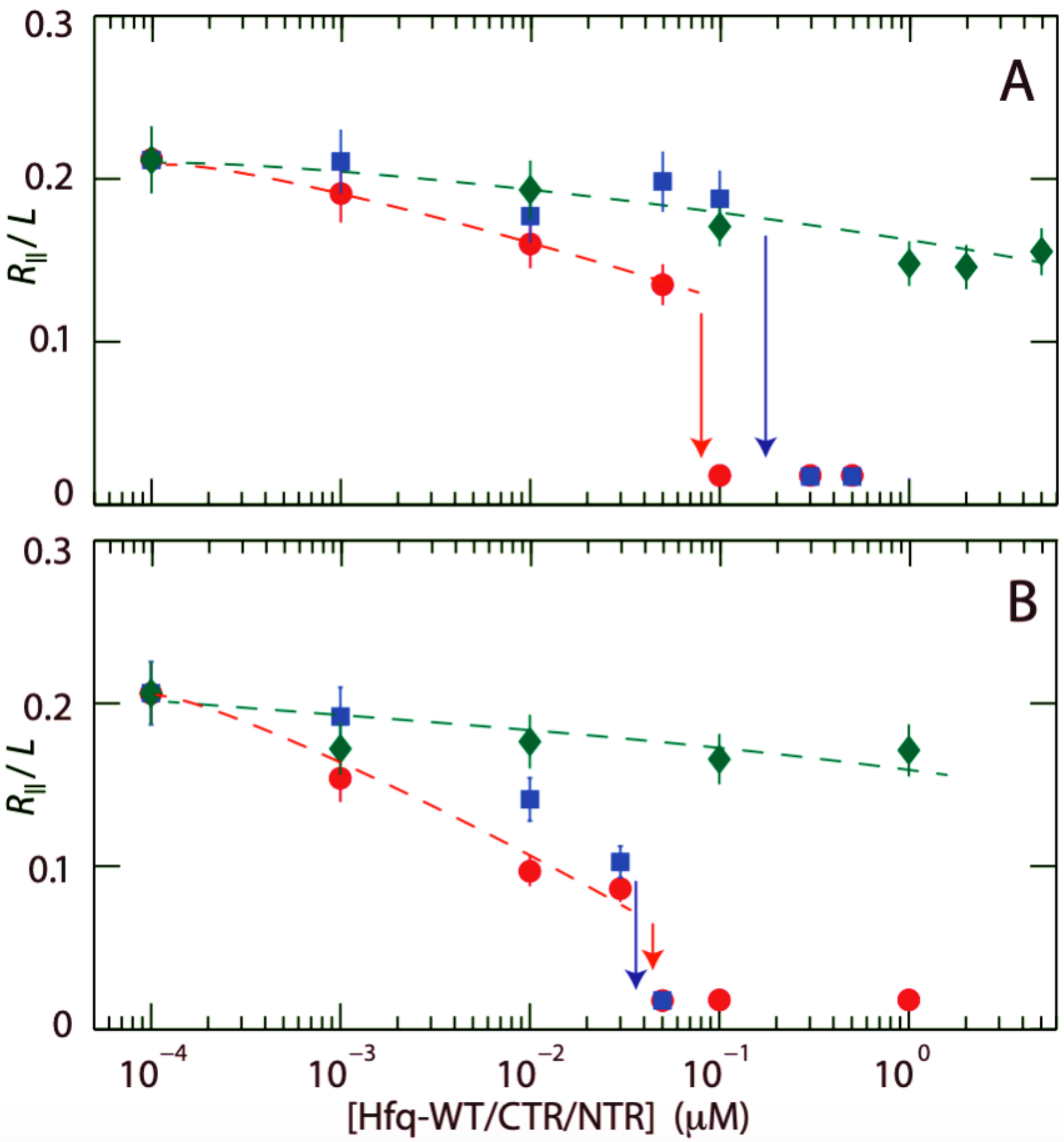

Supplementary figure S2 : Effects of Hfq-CTR on DNA (dA:dT)59 in the presence of $DsrA_{core}$: Blue: DNA alone ; Red: CTR+DNA; Green: CTR+DNA+$DsrA_{core}$ 50 µM.

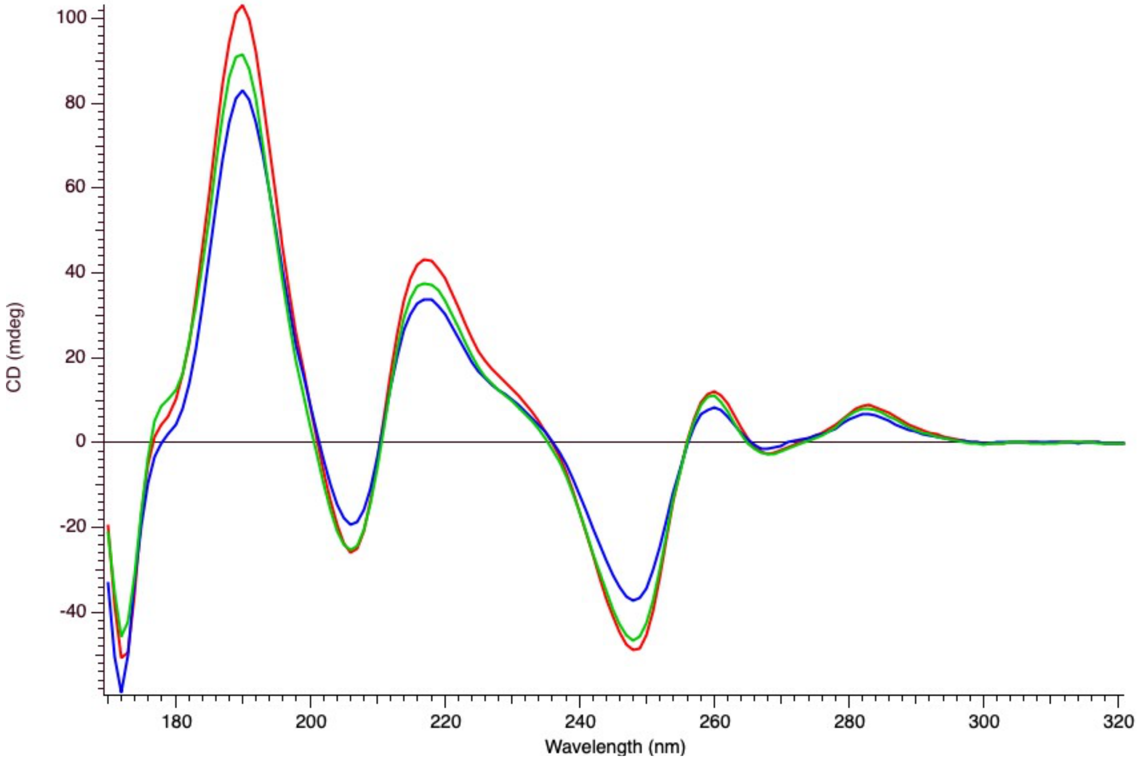

References

(1) Jiang, K.; Zhang, C.; Guttula, D.; Liu, F.; van Kan, J. A.; Lavelle, C.; Kubiak, K.; Malabirade, A.; Lapp, A.; Arluison, V.; et al. Effects of Hfq on the conformation and compaction of DNA. *Nucleic Acids Res* **2015**, *43* (8), 4332–4341. DOI: 10.1093/nar/gkv268.

(2) Malabirade, A.; Jiang, K.; Kubiak, K.; Diaz-Mendoza, A.; Liu, F.; van Kan, J. A.; Berret, J. F.; Arluison, V.; van der Maarel, J. R. C. Compaction and condensation of DNA mediated by the C-terminal domain of Hfq. *Nucleic Acids Res* **2017**, *45* (12), 7299–7308. DOI: 10.1093/nar/gkx431.
